# Genome Editing in Livestock, Complicity, and the Technological Fix Objection

**DOI:** 10.1007/s10806-021-09858-z

**Published:** 2021-05-11

**Authors:** Katrien Devolder

**Affiliations:** Oxford Uehiro Centre for Practical Ethics, University of Oxford, Oxford, UK

**Keywords:** Genome editing, Livestock, Crispr-Cas9, PRRS, Technological fix, Factory farming, Complicity, Ethics

## Abstract

Genome editing in livestock could potentially be used in ways that help resolve some of the most urgent and serious global problems pertaining to livestock, including animal suffering, pollution, antimicrobial resistance, and the spread of infectious disease. But despite this potential, some may object to pursuing it, not because genome editing is wrong in and of itself, but because it is the wrong kind of solution to the problems it addresses: it is merely a ‘technological fix’ to a complex societal problem. Yet though this objection might have wide intuitive appeal, it is often not clear what, exactly, the moral problem is supposed to be. The aim of this paper is to formulate and shed some light on the ‘technological fix objection’ to genome editing in livestock. I suggest that three concerns may underlie it, make implicit assumptions underlying the concerns explicit, and cast some doubt on several of these assumptions, at least as they apply to the use of genome editing to produce pigs resistant to the Porcine Reproductive and Respiratory Syndrome and hornless dairy cattle. I then suggest that the third, and most important, concern could be framed as a concern about complicity in factory farming. I suggest ways to evaluate this concern, and to reduce or offset any complicity in factory farming. Thinking of genome editing’s contribution to factory farming in terms of complicity, may, I suggest, tie it more explicitly and strongly to the wider obligations that come with pursuing it, including the cessation of factory farming, thereby addressing the concern that technological fixes focus only on a narrow problem.

## Genome Editing in Livestock

As the world population grows and developing countries become more affluent, demand for meat, dairy and eggs is expected to increase significantly in the coming decades, especially in lower and middle-income countries (Robinson & Pozzi, 2011). Attempts to meet this demand will, in the absence of mitigating measures, exacerbate global problems caused by the intensive production of these animal products: non-human animal suffering, pollution, antimicrobial resistance, and the spread of non-human animal and human infectious disease (Goodland & Anhang, 2009; Robinson & Pozzi, 2011).

Genome editing, using recently developed nuclease guided technologies, in particular the CRISPR-Cas9 system, could potentially be used in ways that help tackle some of these problems while also maintaining agricultural productivity. Genome editing could, for example, confer disease resistance in livestock (Proudfoot et al., 2019). Researchers have used CRISPR-Cas9 to successfully produce pigs that are resistant to Porcine Reproductive and Respiratory Syndrome (PRRS), which causes reproductive failure, pneumonia and increased mortality in pigs, and results in enormous financial losses for farmers (Burkard et al., 2018). Researchers are also investigating the use of genome editing to produce pigs that are resistant to other diseases, including the porcine epidemic diarrhoea virus and the transmissible gastro-enteritis virus (Proudfoot et al., 2019), and to produce chickens that are resistant to one subgroup of the avian leucosis virus or to avian influenza (Lee et al., 2017). At the time of writing, however, no such gene-edited chickens have yet been produced.

In some cases, conferring disease resistance in livestock could have clear health benefits for humans as well. For example, the virus causing PRRS compromises pigs’ immune systems, which results in severe secondary infections that are mostly bacterial. The production of PRRS-resistant pigs could help reduce the need for antibiotics, and thus reduce the risk of antimicrobial resistance in humans. Likewise, producing gene-edited chicken or pigs immune to influenza would not only help prevent the edited chickens or pigs from contracting influenza, it would also reduce the risk of a human influenza pandemic (Proudfoot et al., 2019).^1^

Other potential uses of genome editing would better adapt livestock to the environment that farmers, and human societies more generally, have created for them. For example, scientists recently bred Angus cattle that carry a heat-tolerance gene called ‘Slick’ (Davis et al., 2017), an alteration that might help them to survive in a world affected by global heating. Scientists have also successfully produced polled (i.e., hornless) dairy cattle using genome editing (Carlson et al., 2016; Schuster et al., 2020). Most breeds of dairy cow have horns, which can cause serious injuries to the famers handling them and to other animals. To prevent such injuries, farmers routinely use painful procedures to remove horns or horn buds. Producing polled cattle through genome editing could replace routine dehorning or disbudding practices (Schuster et al., 2020).

Finally, another example of how genome editing could be used in a way that would arguably be good for livestock, is by reducing the need for culling of around 6 billion of unwanted male chicks per year in the egg industry (Ryan, 2019). The Commonwealth Scientific and Industrial Research Organisation (CSIRO) is currently undertaking proof-of-concept research to show that CRISPR-Cas9 could be used to produce chickens that express a fluorescent marker on the sex chromosomes. When the eggs are illuminated with a fluorescent light, the male embryos fluoresce, and are not put in an incubator (CSIRO, 2020). This could prevent animal suffering and diminish the need for labour-intensive sorting of hatched chicks.^2^

Thus, it seems that genome editing in livestock could enable a win–win situation for humans and livestock and could help resolve some of the most urgent and serious global problems pertaining to livestock. But despite this potential, it is likely that many would object to pursuing it, not because genome editing is wrong in and of itself, but because it is the wrong kind of solution to the problems it addresses: it is merely a ‘technological fix’ to a complex societal issue.

Concerns about technological fixes are not new, and have been adduced against other technological solutions, including in agriculture. For example, in his essay, ‘The Myths of Agricultural Biotechnology’, Miguel Altieri, an agroecologist from the University of California writes that,

By challenging the myths of biotechnology, we expose genetic engineering for what it really is; another technological fix… aimed at circumventing the environmental problems of agriculture (which themselves are the outcome of an earlier round of technological fixes) without questioning the flawed assumptions that gave rise to the problems in the first place (Altieri, 2000.^3^)

And on Greenpeace’s website, we can read that,

GE [genetically engineered] ‘Golden’ rice does not address the underlying causes of VAD [vitamin A deficiency], which are mainly poverty and lack of access to a healthy and varied diet. This GE rice is a technological fix that may generate new problems (Greenpeace Southeast Asia, 2013).

Similar worries are likely to be raised regarding the use of genome editing in livestock.

An organic pig farmer expressed this concern about genome editing to prevent the spread of PRRS:

If gene editing is being used for disease resistance and it is not encouraging companies to change the way they keep their pigs so they don't get disease in the first place, then it becomes a problem rather than a solution (Ghosh, 2018).

Yet though these concerns may have wide intuitive appeal, it is not always clear what, exactly, the moral problem is supposed to be. The aim of this paper is to formulate and shed light on the ‘technological fix objection’ to genome editing in livestock by disentangling and evaluating the various concerns that may underlie it. Only if we better understand the objection, can we determine how much weight to give it, and how to avoid it.

In what follows, I suggest that three concerns may underlie the technological fix objection to genome editing livestock, make implicit assumptions underlying the concerns explicit, and cast some doubt on several of these assumptions, at least as they apply to the use of genome editing to produce pigs resistant to the Porcine Reproductive and Respiratory Syndrome (PRRS) and polled cattle (two applications of genome editing that are currently technically feasible). I then suggest that the third, and most important, concern could be framed as a concern about complicity in the problematic practice of factory farming. I suggest ways to evaluate this concern, and to reduce or offset any complicity in factory farming. Thinking of genome editing’s contribution to factory farming in terms of complicity, may, I suggest, tie it more explicitly and strongly to the wider obligations that come with pursuing it, thereby addressing the concern that technological fixes focus only on a narrow problem. It confronts us with the fact that most of our actions are connected to wrongful practices but that this doesn’t necessarily mean we shouldn’t pursue them. We can and must think of pragmatic ways to reduce or offset our complicity so that, overall, we can expect to produce better outcomes than if we didn’t pursue those actions.

## The Technological Fix Objection

What might it mean if one says genome editing in livestock is a ‘technological fix’, and thus, ‘the wrong kind of solution’? And would this objection be well-founded if raised against genome editing in livestock? Most people accept at least some technological fixes to human problems, such as filters in smokestacks of chimneys in polluting factories, or cholesterol lowering drugs in people with high cholesterol due to genetic or dietary factors. What is the morally relevant difference between these fixes and using genome editing in livestock? In what follows, I begin to address this question focussing especially on the examples of using genome editing to prevent PRRS in pigs, and to prevent painful dehorning or disbudding procedures in dairy cattle. I use these examples as case studies, in order to make the issues more concrete, but concede that, by focussing on them, I may miss other issues that could be raised by other applications of genome editing in livestock. How far the issues I discuss extent to other applications is a question I must leave open.

### Failing to Tackle the Cause of the Problem

One of Greenpeace’s concerns about golden rice is that it doesn’t address the underlying cause of vitamin A deficiency; it doesn’t address poverty and lack of access to a healthy and varied diet. It is a technological fix: a technological solution to a problem that is ultimately social and political in nature. As Dane Scott explains in his book on food, genetic engineering, and technological fixes in the context of agriculture, one aspect of the technological fix objection to golden rice is that it fails to focus on the root social and political causes of the problem (Scott, 2011, 81).^4^ Likewise, some who advance the technological fix objection against genome editing in livestock may be concerned that it merely superficially tackles the symptoms, without addressing the root cause of the problem, such as our disregard for animal welfare and the existence of economic and social structures that push us towards intensive farming practices, in particular factory farming.

Take, for example, genome editing to produce pigs resistant to PRRS (which causes reproductive failure, pneumonia and increased mortality). The concern could be that even if it manages to reduce the spread of PRRS among pigs, it will fail to address the root cause of the problem: that large numbers of pigs, with compromised immune systems,^5^ are confined in small spaces in factory farms, enabling the disease to spread easily and quickly. Likewise, one could object to genome editing to produce polled dairy cattle on the grounds that it doesn’t tackle the underlying problem: the fact that dairy cattle are housed in confined spaces.

It is indeed true that genome editing to produce PRRS-resistant pigs or polled dairy cattle wouldn’t address these underlying problems, but does this give us a good reason to eschew these applications of genome editing? It might if our sole goal is to target the underlying problems. But if our goal—or one of our goals—is the narrow one of ‘reducing the risk of a PRRS outbreak among pigs in confined spaces’ or ‘reducing the risk of injuries caused by horned dairy cows in confined spaces’, it is not immediately clear why genome editing would be problematic even if it doesn’t address the root causes of the problem. Many widely accepted technological fixes are aimed at solving a narrow problem without addressing the root causes of the problem. The goal of cholesterol lowering drugs is to reduce cholesterol levels, not to tackle the root causes of the problem, such as genetic predispositions, unhealthy eating habits and lack of exercise, and the social and political factors that influence lifestyle choices. So even if it is correct that genome editing in livestock to prevent PRRS or painful dehorning or disbudding practices merely treats the symptoms, not the underlying cause of the problem, this may not be all that problematic as long as one clearly defines the goal of the genome editing.

But there may be a further, related, and more serious concern that motivates the technological fix objection to genome editing in livestock.

### Maintaining the Cause of the Problem

Perhaps the concern is not only that genome editing fails to address the cause of the problem it is trying to fix, but that it might actually also *sustain* it. Recall the organic pig farmer’s concern about genome editing to prevent the spread of PRRS:

If gene editing is being used for disease resistance and it is not encouraging companies to change the way they keep their pigs so they don't get disease in the first place, then it becomes a problem rather than a solution (Ghosh, 2018).

The idea here is that genome editing to prevent PRRS *becomes* the problem as it arguably contributes to the causes of the problem it is meant to fix: the way pigs are housed. A similar concern was expressed by some participants of a roundtable discussion on genome editing in livestock, co-hosted by A Bigger Conversation (Beyond GM) and Compassion in World Farming, in London on 18 June 2019 (A Bigger Conversation, 2019). Some participants stressed that genome editing should not be used “to address diseases that primarily result from keeping animals in stressful, crowded conditions. Such diseases should be tackled by improving housing, husbandry and hygiene” (A Bigger Conversation, 2019, 5). These participants did not object to all applications of genome editing in livestock, indicating that their main concern was not that genome editing doesn’t tackle the root causes of the problem it is trying to fix (almost none of the applications of genome editing in livestock do). Rather, the concern is that there is something problematic about us *creating and sustaining* the problem that we are trying to fix through genome editing. Presumably, a similar concern could be adduced against the use of genome editing to produce polled dairy cattle: doing so would arguably contribute to the problem of cramped housing conditions; if the cows were given more space, less injuries would occur, and there would be no need for genome editing in the first place.

This concern, insofar as it applies to the use of genome editing to prevent PRRS and to painful dehorning and disbudding procedures, is grounded in at least two implicit assumptions that we should make explicit in order to evaluate it.

A first implicit assumption is that these applications of genome editing will remove incentives to address the root cause of the problem: the lack of space.^6^ Presumably the thought is that if pigs are resistant to PRRS, farmers will (continue to) keep pigs close together, as they will not have to worry about the risk of the disease spreading between animals. Likewise, it could be argued that with polled dairy cattle, farmers will continue to keep dairy cattle in confined spaces, as they won’t have to worry about injuries caused by horns. Whether this will be the case is an empirical question that I cannot answer. However, one reason to think that the predicted effect may not occur in the case of PRRS-resistant pigs is that, currently, risk of infectious disease does not seem to significantly affect how pigs are housed in industrial settings; animals are frequently housed extremely close together despite not being resistant to disease. Thus, though in an ideal world, pigs would be given more space to prevent infectious disease, it seems that current realities militate in a different direction. Moreover, the genome edited pigs could still suffer from other infectious diseases, reducing the risk, if there is any, that genome editing to prevent PRRS would remove incentives to improve housing conditions for pigs. Thus, for this application of genome editing, it may not be all that likely that it will significantly affect how pigs are housed, and thus, that it will move us further away from farms where pigs are given more space. What about genome editing to produce polled dairy cattle? Will this remove incentives to adapt the cattle’s environment so as to avoid potential injuries? Again, this effect could indeed occur, though there is some reason to doubt that the effect will be significant. Farmers often have very strong preferences about whether they want to breed horned or dehorned cattle. Many farmers strongly dislike dehorning or disbudding cows because of the pain this causes to the animals (Sandøe et al., 2019), but this typically doesn’t make them switch to a different housing system to allow cows to keep their horns (The Dairy Site, 2010). So, it is not clear whether the option of gene-edited polled cattle would have a significant effect on an already existing resistance to switch to a different housing system. On the other hand, it is not unthinkable that the availability of polled cattle will have *some* impact on the way dairy cattle is housed; it would clearly provide a more satisfactory solution (incl. for the farmers) compared to dehorning and disbudding practices. Thus, the concern about genome editing removing incentives to switch to alternative housing systems may be somewhat weightier in the case of polled cattle, than it is in the case of PRSS-resistant pigs.

In any case, my point is that we should make the implicit assumptions underlying this aspect of the technological objection against genome editing in livestock explicit, so that, we can investigate it on a case-by-case basis, i.e., per application of genome editing in livestock (i.e., will application X sustain the cause of the problem it is meant to tackle?).

The second implicit assumption in the concern expressed by the organic pig farmer (and, presumably, by some of the participants of the roundtable discussion) is that giving pigs more space is, *qua* solution to the spread PRRS, morally preferable to genome editing. The quote gives the impression that the cause of PRRS is keeping pigs in close confinement, but that may be somewhat misleading. PRRS is highly infectious and can be transmitted directly and indirectly between farms and other sites in various ways. Some studies suggest that the virus causing PRRS is wind-borne (Arruda et al., 2019). Thus, PRRS would probably still occur if pigs were given more space (though would spread less quickly). But even if PRRS only affected animals housed closely together, giving animals more space may be only one among other ways of preventing it. A more neutral way of presenting the situation would, then, be to say that PRRS exists, and there are (at least) two potential approaches to stopping its spread: (i) giving pigs more space (and providing less stressful housing conditions in general), and (ii) genome editing to produce pigs resistant to PRRS.^7^ Likewise we could say that dairy cows’ horns can cause injuries, and there are (at least) two potential approaches to prevent this: (i) giving dairy cattle more space, and (ii) genome editing to produce polled dairy cattle. Those appealing to the technological fix objection against genome editing to produce PRRS-resistant pigs or polled dairy cattle assume that (i) is morally preferable to (ii). But why? Suppose that both are equally effective at preventing the spread of PRRS, or injuries caused by horns. Why is it better to keep pigs or cows further apart?^8^

Simply claiming that (i) is preferable because (ii) is a technological fix won’t do as this would amount to circular reasoning and would beg the question against a proponent of such fixes. We were trying to explain why technological fixes are problematic, and it won’t do to appeal to the fact that they are technological fixes. So, a further explanation is needed for why giving more space to pigs or cows is preferable to genome editing, assuming that both are effective ways to stop the spread of PRRS or prevent injuries. Perhaps the most plausible way to explain why giving more space to the animals is preferable to genome editing is by referring to the side-effects of both actions. Keeping pigs and cows further apart should be preferred because it is better for them not only by virtue of preventing PRRS in the case of pigs, and injuries in the case of cows, but in other respects as well. For instance, giving them more space is, independently of its effects on PRRS and injuries, better for their wellbeing. Thus, giving pigs and cows more space provides a solution to more than one problem, whereas genome editing arguably would not have any such positive side-effects. Indeed, it may have a cost to the animals’ welfare, as the development of the technology requires potentially harmful scientific experiments on them (e.g., experiments resulting in late term abortions).

However, we should also not forget that giving more space to livestock may have some undesirable side-effects as well. More land will be needed, which, assuming the number of pigs and cows remains constant,^9^ means less land for growing crops that are more nutritiously efficient, less threatening to animal wellbeing, and less conducive to global heating and the environment. These disadvantages also need to be taken into account when weighing costs and benefits of the use of genome editing to prevent PRRS and injuries caused by horns. Thus, in order to determine whether improving housing conditions is morally preferable to genome editing, we need to know more about, and compare, the expected side effects of the various alternative solutions to preventing PRRS in pigs or injuries in the case of cows.

Keeping pigs and cows further apart could also be considered morally preferable because technological solutions are thought to produce a kind of technological dependence. So-called ‘neo-luddites’, for example, oppose technologies that they believe to be ‘threats to human lives and communities’, and genetic modification is considered one such a threat. They favour instead technologies “that foster independence from technological addiction and promise political freedom, economic justice, and ecological balance” (Glendinning, 1990). So, one thought here seems to be that genome editing will make us increasingly dependent on technological fixes. Of course, the question, again, is why that would be bad. An oft-adduced answer relates to another concern that neo-luddites, and others, have expressed about technological fixes: they generally are thought to be to the disadvantage of the more vulnerable, for example, the poorest inhabitants of developing countries. This is because the fixes are typically tied to powerful biotech companies, who may exert too much control over local farming and agricultural practices, threatening traditional practices that are more widely accessible.

This is an important concern, as is also illustrated by the case of the ‘Enviropig’.^10^ In 1999, researchers at the University of Guelph in Ontario produced a line of genetically engineered pigs as a technological fix for global phosphorus pollution caused by the high concentration of phosphorus in pigs’ manure. The genetically engineered Enviropig’s manure can contain up to 75% less phosphorus than that of normal pigs (Golovan et al., 2001). However, as Scott, (2018) points out in his detailed analysis of the Enviropig case, there are reasons to think that the Enviropig was primarily created to increase the economic efficiency of the Canadian hog production industry, rather than to decrease worldwide phosphorus pollution as it was not designed for countries where a low-polluting pig could have the greatest impacts to mitigate phosphorus pollution. For instance, the developers of the technology used a breed that is not native to those regions. Moreover, intellectual property rights, costs associated with user technology fees, and patent protection, would have hampered widespread use of the Enviropig in low- income countries (Scott, 2018, 104–110).

Interestingly, there is some reason to believe that concerns about an increasing dependence on technological fixes (and perhaps, as a result, concerns about widespread access) may be weaker in the case of genome editing to produce polled dairy cattle than in the case of genome editing to produce PRRS-resistant pigs. More is to be said about this, but it is worth pointing out that genome editing to produce polled cattle would only be a temporary measure, because over time, the service sires would be polled merely based on inheritance from their parents (Mueller et al., 2019).

In any case, my point here is again that when the technological fix objection is adduced against genome editing in livestock, we need to make the implicit assumptions explicit, to investigate whether they are correct, but also to help us identify the various concerns that motivate the technological fix objection. Objecting to genome editing merely because it is a technological fix can be a rather opaque claim that may hamper further debate about pragmatic solutions to tackle the most important concerns underlying the objection (e.g., how to ensure genome editing is developed so that it does most good globally?). What my analysis of the technological fix objection against genome editing in livestock so far suggests is that, when applied to the prevention of PRRS in pigs and of disbudding and dehorning practices in dairy cows, it may give us a reason to be suspicious of the way the technology will be implemented (e.g., in a way that promotes injustice) but it is not a devastating objection to the application of the technology itself (though admittedly, my analysis is not complete, so this is a tentative conclusion). Moreover, my (admittedly incomplete) analysis suggests that some of the underlying concerns may be weaker against genome editing to produce polled cattle (e.g. concerns about increasing dependence on technological solutions) whereas other concerns may be more powerful (e.g., genome editing to produce polled cattle may more likely take away incentives to improve housing conditions, compared to genome editing to produce PRRS-resistant pigs).

But yet another problem persists: even if genome editing to produce PRRS resistant pigs or polled cattle may not remove incentives to provide more space to animals, arguably, it may help to sustain factory farming itself and the wider problems that it causes.

### Maintaining Factory Farming and the Wider Problems it Causes

A third concern then can be thought of as a variant of the second but focusses on a particular kind of negative side-effect: in using a technological fix to solve a narrow problem (e.g., of the prevention of PRRS in pigs in confined spaces) we move further away from the optimal solution to the wider problem of the harms caused by factory farming. It may be thought that even if genome editing to prevent PRRS doesn’t have any direct effect on housing conditions of pigs, it nevertheless legitimises and facilitates factory farming in general, thereby helping to maintain it, and that this makes it so morally problematic. Scott, (2018, 112) points out that one criticism of technological fixes is that they frequently are conservative-they preserve rather than replace a flawed system. Again, there are implicit assumptions here that we need to make explicit.

The first is that the cessation of factory farming, is *qua* solution to the aforementioned global problems, morally preferable to genome editing in livestock. The second one is that genome editing in livestock moves us further away from this morally preferable solution. And the third one is that this provides a decisive reason to forego genome editing in livestock.

Let’s begin with the first assumption, that the cessation of factory farming is a better solution to genome editing. There are very good reasons against factory farming as we currently know it. It causes immense animal suffering, pollution, global heating, antimicrobial resistance and infectious disease. Thus, let us assume abandoning factory farming is the morally best option available. Then we need reasons for thinking that genome editing in livestock will move us further away from it. This is the second assumption underlying this version of the technological fix objection.

Whether genome editing would slow down the transition to the cessation of factory farming is an empirical question that is difficult to answer. Perhaps it could do so by taking away incentives to move to alternative agricultural systems. Though there is some chance that any measure to improve animal wellbeing—even, say, providing larger cages for chickens—might delay the abolition of factory farms, the risk seems greater with genome editing. It could be argued that providing larger cages goes more against the spirit of factory farming. It comes closer—though admittedly not much closer—to what it would be like if animals were not contained in factory farms. Moreover, the cost of adopting such measures in factory farms is significant. Genome editing could be ‘accused’ of going along with the practice of factory farmingit facilitates factory farming; it makes it more efficient. This may entail more risk that it will indeed slow down a transition to alternative agricultural practices. However, the effects of genome editing on incentives to move away or towards factory farming are uncertain, whereas the potential advantages of genome editing to prevent PRRS are more certain.

Finally, there is also a third assumption that we need to make explicit, namely that if there is a high risk that genome editing in livestock would remove us somewhat further from abandoning factory farming, this is so problematic that it provides a conclusive reason not to pursue it. But is this plausible? After all, the same reasoning would suggest it was wrong to improve the welfare of slaves in the United States in the eighteenth and nineteenth century, which may have delayed the abolition of slavery. Even if it did delay abolition, we surely think that it was morally justifiable, even obligatory, to improve the slaves’ lives whenever that was feasible.

If the stakes are high, it may be acceptable, or morally obligatory, to take the risk that genome editing in livestock will slow down the transition to a better solution. But even if we accept this general conclusion, how do we determine whether a particular application of genome editing would be permissible?

In the discussion above, I noted at several points that we need to weigh the possible costs of genome editing (for example, those due to delaying the cessation of factory farming) against the benefits (for example, in the form of improved animal wellbeing in the short and medium term). This suggests that one approach would be to simply perform a cost–benefit analysis for each potential application of genome editing, and proceed if and only if the expected benefits outweigh the expected costs. However, one problem with this approach is that it assumes that consequences are all that matter. Yet some might deny this. For example, some might claim that it is more problematic to intentionally contribute to the maintenance of factory farming than to unintentionally contribute to it, even if the consequences are the same. We need some way of taking into account such factors. I suggest that a useful approach might be to think in terms of moral complicity.

## Complicity in Factory Farming

The concept of moral complicity captures the idea that one can do wrong by being associated, in some way, with the wrongdoing of other individuals or of a collective of which one is a part. A standard case of complicity is that of a getaway driver being complicit in a bank robbery. There are various theories regarding when one’s association with others’ or a collective’s wrongdoing makes one wrongfully complicit in it. Non-causal accounts of complicity may find one’s involvement wrong regardless of whether it makes any difference to what in fact happens. On some non-causal accounts, one can even become complicit in past wrongs. For example, a scientist who uses data acquired by Nazi doctors through unethical medical experiments on concentration camp inmates may thereby become complicit in these atrocities. By contrast, on causal accounts of complicity, for someone to be complicit in others’ wrongdoing one has to have an (expected) effect on it.^11^

Complicity is complex, and not black and white. For the purpose of this paper, I will assume a causal account of complicity, because this type of account is widely accepted, and more easily allows for degrees of complicity (which is an advantage for any account of complicity). However, a similar approach to the one I will suggest,could be taken with at least some non-causal accounts of complicity.^12^ On a causal account of complicity, one can distinguish degrees of complicity, and these differ in their wrongness.^13^ To what extent one is wrongfully complicit in the principal wrongdoing will typically depend on several factors. One such factor is the badness of the principal wrong in which one is complicit. It is obviously less bad to be complicit in the theft of a few apples than to be complicit in Nazi medical experiments. How seriously wrong is factory farming? There is increasing agreement on the fact that it is a grave moral wrong, because of the significant harm to billions of animals, the risk of infectious disease it creates, the negative environmental impact and so on (Harari, 2014; Singer & Mason, 2006). Given that alternative agricultural systems could feed the world without causing so much harm, it is very difficult to justify factory farms. If factory farming is indeed a serious wrong, this suggests that complicity in it is also significantly wrong.

A second important factor is the nature of one’s contribution to the wrong. Determining this is a complex matter, but it is possible, for example, to differentiate between tighter and looser types of contribution. Take a railway heist. Each train robber of Butch Cassidy’s gang ‘The Wild Bunch’ was heavily complicit in the robberies because each robber’s action was partly constitutive of each robbery (even if every robber was on their own inessential for the robbery to succeed). However, many complicit acts are more loosely connected to the principal wrongdoing (e.g., providing the weapons for The Wild Bunch, providing useful information to them to facilitate the robberies, covering up de robberies, and so on). How seriously wrong one’s complicity is may, among other things, depend on whether one’s contribution to the wrong is constitutive or causal, how much difference one makes to the likelihood or wrongness of the wrong, how likely it is to be essential for the wrongdoing to occur, and how proximate it is, in the causal chain, to the wrongdoing (i.e., how many other things in the causal chain are necessary for the wrongdoing to occur; the fewer, the higher the degree of complicity).

Those pursuing genome editing, say, to prevent PRRS in pigs, can be expected to contribute somewhat to the continued existence of factory farms. They facilitate factory farming as they prevent enormous economic losses to factory farms. As mentioned earlier, they are increasing the efficiency of the practices of factory farms to some extent and in a way that, for example, providing more space for pigs wouldn’t. On the other hand, it is not as if all factory farms would cease to exist if genome editing to prevent PRRS were not pursued. One reason is that the political inertia to move away from factory farms has been shown to be very strong, so genome editing in livestock to prevent PRRS is unlikely to do any more than slightly strengthen than inertia. Thus, though this application of genome editing can be expected to somewhat contribute to factory farming, it is doubtful that it will make a large difference to either the likelihood or wrongness of the wrong, and it is causally quite remote from its continued existence, all factors which tend to make any complicity in it less morally problematic. A different conclusion may result from other applications of genome editing in livestock though. For example, genome editing to prevent a disease that was clearly solely caused by the maltreatment of animals in factory farms, would arguably involve a greater degree of complicity, because, in addition to diminishing the costs for the farmers of persisting with factory farming, it arguably also sends a message that it is fine to treat animals in ways that (would) cause this disease. This message will likely help maintain such immoral practice). It would be more akin to doctors assisting in death penalties (assuming here death penalties are wrong)—they would also help the particular victim but would thereby become complicit in an unjust criminal justice practices, which is bad for other convicted individuals.

A third factor that determines the moral weight of one’s complicity is one’s intention. You are more culpable for your complicity if you have the same bad intention as the principal wrongdoer. For example, if you give a loaded gun to Sonja, so she can kill Bill, your complicity in Bill’s murder is morally worse than if you had unintentionally left your gun in an unlocked drawer, Sonja found it, and killed Bill. It is of course not always possible to identify the motives of those supporting or pursuing genome editing in livestock. There are certainly defenders of genome editing who do not share the intentions of factory farmers. Philosopher Adam Shriver is one example; he clearly would like to abandon factory farming (he is a vegan) but has advocated for the use of genome editing to replace factory farmed cows and pigs with ones that have been edited to suffer less from pain (Shriver, 2009). There will also be supporters of genome editing who do share the intentions of factory farming. For example, if scientists financed by a factory farms develop applications of genome editing to increase meat per animal, they may be mostly motivated by the desire to support factory farms. Their complicity in factory farming would therefore be worse compared to that of genome-editing advocates like Shriver.

Thus, supporting or pursuing genome editing in livestock is likely to make one complicit in maintaining factory farming, and this is morally wrong, though there are differences between different applications in the degree, and thus wrongness of this complicity. What are the implications of this?

If we accept that some applications of genome editing in livestock may make us wrongfully complicit in factory farming, it may seem that we should eschew those applications. But there is an alternative. Perhaps we can instead take steps to (partially or fully) negate or offset this complicity. This might allow us to reap the benefits of genome editing without thereby doing (as much) wrong. For example, perhaps we should only pursue a particular application of genome editing if we combine this with introducing e.g., higher taxes for meat, eggs, and dairy from factory farms, or with structural support for the production of lab-grown meat, or alternative and more sustainable farming practices.

Often the pursuit of genome editing in livestock is presented as opposed to or incompatible with all these measures. But that shouldn’t be the case. One can consistently care about both avoiding or reducing complicity in factory farming and reaping short- and medium-term benefits in, for example, animal welfare. And if one cares about both these things, employing genome editing while taking steps to negate or offset complicity may be the optimal strategy.

In doing so, it will be important to consistently present a particular application of genome editing as one small step towards solving some global problem, be it the prevention of pandemics or of animal suffering, i.e., as a partial solution that should be implemented as part of a wider solution to the problems of factory farming.^14^ Referring to genome editing as a technological fix, whether in defence or against it, may therefore not be that helpful as it tends to divert the attention away from the most important concerns that motivate the technological fix objection (such as concerns about equity and justice), and, as a result, from considering and debating the pragmatic solutions we can and should be working towards to address these concerns (e.g., changing the way research is funded,^15^ efforts made to primarily focus on genome editing in livestock where it will have most effect to help tackle global problems).^16^

## Conclusions

What I have referred to as the technological fix objection to genome editing in livestock may be motivated by several concerns: that genome editing doesn’t solve the underlying problem, that it helps to maintain that problem, and that it helps to maintain wider problems, in particular, through its role in maintaining factory farms. I have argued that genome editing will indeed often not solve the underlying problems, but that this in itself is not all that problematic as long as one is explicit about what the target problem is, and one acknowledges that genome editing will only solve that narrow problem. The concern that genome editing will help maintain the cause of the problem it is trying to fix is an important one. I have made explicit the often-implicit assumptions underlying this concern and challenged some of these with regard to genome editing to prevent PRRS in pigs and disbudding and dehorning practices in dairy cows. The main point, however, is that we should make the implicit assumptions underlying this aspect of the technological fixes objection explicit so they can be carefully evaluated. The third concern, I have argued, could be thought of in terms of moral complicity in factory farming. Some applications of genome editing will involve more wrongful complicity in factory farming compared to others. This will determine how easily the complicity can be outweighed by any expected benefits, and to what extent measure should be undertaken to reduce or offset that complicity. Thinking of genome editing’s contribution to maintaining factory farming in terms of complicity has the advantage it ties the technology more clearly and strongly to the moral obligations we have to move to more morally acceptable modes of animal husbandry in the longer term, and thereby addresses the first concern: that technological fixes focus only on the narrow problem.

## Footnotes

1 But note that, to date, no gene-edited chickens or pigs that are resistant to influenza have been produced.

2 There are alternative methods available to prevent the culling of male chicks. For example, in Germany, an egg breeding company uses biomedical markers to distinguish female from male eggs at day 9 after fertilization. Only female eggs are returned to the incubator and hatched (Schulz, 2019). It is, however, beyond the scope of this paper to determine whether any of these alternative methods obviate the need for genome editing to avoid the culling of male chicks.

3 As cited in Scott D.N., (2018), Food, Genetic Engineering, and Philosophy of Technology, p.73.

4 Scott, (2018) identifies three criticisms of technological fixes: (i) they do not solve problems, (ii) they create new problems, and (iii) they preserve, or fix, systems that should be abandoned in favor of better alternatives (see, e.g., p. 88).

5 The pigs’ immune systems are compromised because of the stress they experience as a result of living in confined spaces where they cannot exhibit species-typical behavior, and because of the genetic and pharmaceutical interventions they are subjected to (Martínez-Miró et al., 2016).

6 I am simplifying what the root causes of the problem are in these cases—there may be other aspects that contribute to pigs’ risk of being infected by PRRS and of cows causing injuries with their horns that should be taken into account.

7 Vaccines have also been ineffective for preventing PRRS (Ishii, 2017).

8 Likewise, those *defending* genome editing to prevent PRRS should present reasons for why they think it is preferable to any potential alternatives. These alternatives are also often left out of scientific papers or presentations in defense of genome editing in livestock.

9 I assume here that the number of pigs and cows will remain constant. It is not likely that, in the short-term future, industrial pig and cow farmers will be prepared to reduce the number of animals they breed in order to give them more space. The number of animals farmed could also be reduced if there were enough political will to do so but, at the moment, this political will is not likely to increase either, at least not in the near future.

10 See Scott (2018), pp. 105–110, for valuable insights into the technological fix objection against the Enviropig.

11 For a more detailed overview on various accounts of complicity, see, Devolder K., (2017).

12 Not all non-causal accounts allow for degrees of complicity (e.g., intentional participation accounts have been criticized for the reason that they don’t allow for this, but on Kutz’ influential account of non-causal complicity, for example, one can be more, or less, blameworthy for one’s contributory act depending on the functional role in a collective endeavor, and the degree of intentional overlap (Kutz, 2000, Ch. 5).

13 My analysis is roughly based on the causal account of complicity developed by Lepora and Goodin, (2013), which I think is the most plausible account thus far developed. Lepora and Goodin, (2013, ch. 4, 5) develop a [rough] formula to determine the blameworthiness of an accomplice. They argue that this is a function of (i) the badness of the principal wrongdoing, (ii) the extent to which the agent is responsible for her contribution to that wrongdoing (for example, perhaps the agent didn’t know, and could not have been expected to know that the wrongdoing would occur), (iii) the essentiality, centrality and proximity of the causal connection between one’s contribution and the wrongdoing, and (iv) whether the agent shares the intentions of the principal wrongdoer (if so, this is an aggravating factor).

14 This is consistent with some empirical studies that found that lay publics are cautiously optimistic about some applications of genome editing in animals but thought that these should be considered as part of a package of solutions for global challenges, and that there should be equity of access to the technologies (Van Mil et al., 2017).

15 One of the major themes of Scott’s book (2011) is the need to take greater responsibility for directing research and development of emerging technologies. Scott stresses the need to change the funding system for research, for example by creating a ‘pay for performance’ system for technologies that make measurable progress in addressing specific global environmental problems like phosphorus pollution (Scott, 2018, p. 110, and Chapter 2).

16 That we should focus on these concerns is also corroborated by the important work of de Graeff et al., (2019), which provides a review of ethical reasons for and against genome editing in animals: They write that “the public concern for equity of access to the potential benefits of genome editing technologies, questions about the just distribution of governmental funding of genome editing compared with other investments, and concerns about the commercialization of genome editing technologies” are underrepresented in the academic literature. With regard to commercialization, members of the public have raised the worry that businesses could prioritize profitmaking over the public good and could fail to provide a balanced representation of the benefits and risks of these technologies.” (de Graeff et al., 2019, 154).
